# Neuroleptic Malignant Syndrome After Re-introduction of Atypical Antipsychotics in a COVID-19 Patient

**DOI:** 10.7759/cureus.13428

**Published:** 2021-02-18

**Authors:** Eduardo D Espiridion, Valli Mani, Adeolu O Oladunjoye

**Affiliations:** 1 Psychiatry, Drexel University College of Medicine, Philadelphia, USA; 2 Psychiatry, West Virginia School of Osteopathic Medicine, Lewisburg, USA; 3 Psychiatry, West Virginia University School of Medicine, Martinsburg, USA; 4 Psychiatry, Philadelphia College of Osteopathic Medicine, Philadelphia, USA; 5 Psychiatry, Reading Hospital Tower Health, West Reading, USA; 6 Medicine, Drexel University College of Medicine, Philadelphia, USA; 7 Medical Critical Care, Boston Children's Hospital, Boston, USA

**Keywords:** covid 19, antipsychotic medication, altered mental state, neuroleptic medications, neuroleptic malignant syndrome

## Abstract

We present a case of neuroleptic malignant syndrome (NMS) in a 46-year-old white female from a state psychiatric hospital who also tested positive for coronavirus-2019 (COVID-19) (severe acute respiratory syndrome coronavirus, SARS-CoV-2) infection after re-introduction of her home antipsychotics medication. She presented with confusion and altered mental status likely secondary to delirium from COVID-19 infection. Clozapine and risperidone were initially held on admission and restarted after continuing agitation on day two. She began to have increased restlessness with rising creatinine kinase (CK) levels, peaking on day seven with sudden fever, hypertension, and tachycardia. The diagnosis of NMS was confirmed, antipsychotic medication was held, and appropriate treatment was administered. The mechanism explaining the occurrence of NMS in COVID-19 patients is still unclear, but COVID-19 infection may be a risk factor for this presentation. The mechanism of SARS-CoV-2 as a risk factor for NMS is still uncertain and needs to be investigated further. However, if their infection status is known, patients should be given neuroleptics with caution and carefully considered for the development of this rare condition.

## Introduction

Neuroleptic malignant syndrome (NMS) is a rare life-threatening neurological emergency associated with the use of antipsychotic agents, commonly first-generation or typical neuroleptics [1]. It is characterized by altered mental status, rigidity, fever, and autonomic instability. Coronavirus-2019 (COVID-19), on the other hand, causes flu-like symptoms and acute respiratory distress syndrome but it is now recognized to have a variety of neurological manifestations including altered mental status, impaired consciousness, delirium, encephalopathy, and seizures [2]. Our patient who was infected with COVID-19 was also susceptible to NMS, given her history of antipsychotic use. There are two established case reports of NMS in patients with COVID-19 infection [3-4]. However, our patient was diagnosed with COVID-19 infection while in the state psychiatric hospital, where they do weekly testing to their residents. She initially had fever, chills, fatigue, anosmia, and headaches. There were no initial complaints of rigidity. However, because she is on antipsychotics, an NMS diagnosis was considered in the differential diagnoses. This patient has a long psychiatric history and multiple prior psychiatric hospitalizations. She was diagnosed with schizoaffective disorder when she was 19 and has been stable on the combination of clozapine and risperidone for more than a decade. Her antipsychotics were initially held then restarted after continuous agitation. Over the next couple of days, the patient’s mental status worsened and she developed NMS. 

## Case presentation

History of present illness

A 46-year-old white female with a past medical history of schizoaffective disorder, seizures, hypertension and hypothyroidism, presented to the ED from a state psychiatric hospital with altered mental status. She presented with a one-day history of a head injury after hitting her head against a wall status post manic episode. The staff described the manic episode as restlessness, not sleeping, irritable, impulsive, and internal preoccupation. Those symptoms have been observed for years and she had hit her head against the wall on several other occasions in the past five years. The patient was discharged after CT of the head/neck was negative for acute abnormalities. All the previous CT scans of the brain, which were ordered because of her head banging, showed no abnormalities. At this time, she was tested for a COVID-19 infection and found to be positive. She was afebrile with an oxygen saturation of 97% and no respiratory symptoms, so she was discharged back to the state hospital. The state hospital has a unit dedicated to psychiatric patients who tested positive for the COVID-19 infection. On the day of admission, the patient presented with altered mental status, as well as worsening restlessness and agitation. She also presented with autonomic instability, with hypotension and tachycardia, as well as palpitations. Lead-pipe rigidity was not observed on presentation. Notable home medications were clozapine, divalproex, escitalopram, lithium, and risperidone. After arriving at the ED, she received lorazepam 2 mg and ziprasidone 10 mg stat for sedation. At this time, risperidone 4 mg twice daily and clozapine 300 mg daily were held and the patient was admitted for encephalopathy.

Examination

After admission, the patient continued to be confused, disrobing, and unable to respond to commands. She was not responding to verbal redirections. Clozapine 300 mg daily and risperidone 4 mg twice daily were restarted as her home dose because of these symptoms. Over the next few days, she had intermittent periods of restlessness and thrashing of her extremities with disorientation. Prior to this admission, the patient was eating and drinking water but the oral intake became lesser than usual. In addition, the patient was still communicating with staff and her peers. Catalepsy was not observed. Unfortunately, her oral intake became worse and she was combative whenever assessed by the medical team so sedation was required throughout her hospitalization. On hospital day six, she exhibited abnormal vital signs: febrile temperature 103.8°F (reference range: 97-99°F), hypertension 180/89 mmHg (reference range: <120/80 mmHg), tachycardia 138 bpm (reference range: 60-100 bpm), and hypoxemia 91% (reference range: 95%-100%). On physical examination, the patient did not have typical “lead pipe” rigidity; however, her extremities were rigid with passive movement. In addition, she appeared to be responding to internal stimuli with no awareness of her surroundings. That evening, the patient’s fever spiked to 105.9°F and declined to 103.6°F after receiving acetaminophen 650 mg and lorazepam 1 mg. 

Investigation

When the patient tested positive for COVID-19, her chest X-ray showed bilateral basal infiltrates which was thought to be atelectasis. On initial assessment, laboratory findings were only significant for a creatinine kinase (CK) of 303 U/L (reference range: 30-200 U/L), which was attributed to agitation. Creatinine and vital signs were normal. Her CK continued to increase to 550 U/L and then later to 668 U/L. By day six, significant laboratory findings included CK of 1096 U/L, along with creatinine 1.44 mg/dL (reference range: 0.84-1.21 mg/dL) and white blood cell count 13.5 x 109 /L (reference range: 4.5-11 x 109 /L). Other significant laboratory values included hypernatremia 159 mmol/L (reference range: 136-145 mmol/L) and hyperchloremia 122 mmol/L (reference range 96-106 mmol/L). On day seven, CK peaked at 4,842 U/L. Autonomic instability, rigidity, rhabdomyolysis, and leukocytosis, along with altered mental status, in the setting of administration of multiple antipsychotic medications, raised suspicion for NMS. Both clozapine and risperidone were held. Other conditions were considered in the differential diagnoses. Catatonia was ruled out because her detailed psychiatric history did not reveal any episodes of catatonia while she was taking neuroleptics. The absence of mutism and the high levels of CK pointed to NMS. Traumatic brain injury was also considered because of the history of repeated head banging, but the CT scan of her brain showed no acute abnormalities. Serotonin syndrome presents the same way but the elevated CK levels do not occur in this condition. Malignant hyperthermia is another condition ruled out because there was no exposure to halogenated inhalational anesthetics or depolarizing muscle relaxants.

Treatment

All antipsychotic medications were held and the patient was given lorazepam 1 mg as needed. She was transferred to the ICU and intubated for airway protection and to facilitate sedation. Another reason for the intubation was the presence of tachypnea, with a respiratory rate of 20, and labored breathing. At the ICU, she was treated with bromocriptine 2.5 mg per nasogastric tube every 12 hours and dantrolene 100 mg intravenously every six hours for the next five days. Empiric cefepime was started, as sepsis and meningitis were also considered as differentials, but discontinued when lumbar puncture and blood cultures eventually returned negative. Fever, tachycardia, and hypertension had resolved upon supportive care and intubation. CK and creatinine began to downtrend over the next week. Around this time, the CK went down to 242 U/L and her creatinine went down to 0.36 mg/dL. The patient was extubated two weeks later; however, repeat blood cultures were positive for methicillin-susceptible Staphylococcus aureus (MSSA). She remained in the hospital for treatment with cefazolin. Patient was re-evaluated by the psychiatrist, who documented her delirium had improved. Her mood and psychotic symptoms did not exacerbate and remained stable. She was observed to be eating and drinking fluids and she engaged during the evaluations. After completion of her treatment, she was then transferred to the state psychiatric hospital for long-term care.

## Discussion

Evidence shows that in the progression of symptoms for NMS, altered mental status appears first before hyperthermia and autonomic dysfunction in over 80% of cases [5]. This is shown through the patient’s worsening mental status after day two of hospitalization when clozapine and risperidone were re-introduced to her medication regimen. The keys to her diagnosis of NMS were hyperpyrexia and rhabdomyolysis, exhibited by elevated CK and creatinine. She also presented with rigidity and autonomic instability. Hypernatremia and hyperchloremia were secondary to dehydration from spiking fevers. CT scan of the brain did not show any acute abnormalities. This patient had been treated with antipsychotics in a state psychiatric facility for over 20 years and had no prior history of NMS. Patients at the highest risk of NMS are elderly males on typical antipsychotics, such as haloperidol and fluphenazine, due to their potent dopamine antagonism or those who switch to a higher dose of antipsychotic [1].

Some COVID-19 patients without typical symptoms (fever, cough, diarrhea) present only with neurological symptoms [2]. In this case, the patient did not have any respiratory symptoms with only mild atelectasis on chest X-ray on initial presentation. Subsequently, she presented with altered mental status, agitation, and restlessness after infection with COVID-19. Reports of nonspecific central nervous system manifestations of COVID-19 are increasing, including headache, impaired consciousness, delirium, encephalopathy, seizures, and loss of taste and smell due to direct viral infection or inflammation of the CNS [6]. On a cellular level, it has been proposed that severe acute respiratory syndrome coronavirus (SARS-CoV-2) utilizes ACE2 receptors on host cells for its internalization [6]. These receptors are encoded in astrocytes, oligodendrocytes, and neurons, as well as other neurological structures in the brain, leading to the speculation that COVID-19 has the potential to infect neurons and glial cells [6]. It is possible that COVID-19 infection increases the susceptibility of patients prescribed antipsychotics to NMS as an additional risk factor. However, the exact mechanism is still unknown.

All antipsychotics may trigger NMS regardless of the patient's age, gender, or ethnicity. It is a potential side effect of second-generation antipsychotics which includes both clozapine and risperidone [7]. Atypical antipsychotics were assumed to be free from risks of NMS. It should be considered and ruled out if hyperpyrexia occurs during the course of antipsychotic treatment. This patient contracted COVID-19 sometime before she was admitted and her antipsychotics were initially held when first seen but later resumed when she continued to be agitated. Because of the emergence of other issues including elevated CK levels, autonomic instability, rigidity, and severe altered mental state, NMS was considered and her antipsychotics were held. 

## Conclusions

The mechanism explaining the occurrence of NMS in COVID-19 patients is still unclear, but this infection may be a risk factor for the presentation of NMS in this patient. The mechanism of SARS-CoV-2 as a risk factor for NMS needs to be investigated further. However, if their infection status is known, psychiatric patients should be given neuroleptics with caution and carefully considered for the development of this rare condition.
